# Bacterial Findings in Infected Hip Joint Replacements in Patients with Rheumatoid Arthritis and Osteoarthritis: A Study of 318 Revisions for Infection Reported to the Norwegian Arthroplasty Register

**DOI:** 10.5402/2012/437675

**Published:** 2012-10-17

**Authors:** J. C. Schrama, O. Lutro, H. Langvatn, G. Hallan, B. Espehaug, H. Sjursen, L. B. Engesaeter, B.-T. Fevang

**Affiliations:** ^1^Department of Orthopaedics, Haukeland University Hospital, 5021 Bergen, Norway; ^2^Section of Infectious Diseases, Medical Department, Haukeland University Hospital, 5021 Bergen, Norway; ^3^Norwegian Arthroplasty Register, Haukeland University Hospital, 5009 Bergen, Norway; ^4^University of Bergen, Bergen, Norway; ^5^Department of Rheumatology, Haukeland University Hospital, 5021 Bergen, Norway

## Abstract

High rates of *Staphylococcus aureus* are reported in prosthetic joint infection (PJI) in rheumatoid arthritis (RA). RA patients are considered to have a high risk of infection with bacteria of potentially oral or dental origin. One thousand four hundred forty-three revisions for infection were reported to the Norwegian Arthroplasty Register (NAR) from 1987 to 2007. For this study 269 infection episodes in 255 OA patients served as control group. In the NAR we identified 49 infection episodes in 37 RA patients from 1987 to 2009. The RA patients were, on average, 10 years younger than the OA patients and there were more females (70% versus 54%). We found no differences in the bacterial findings in RA and OA. A tendency towards a higher frequency of *Staphylococcus aureus* (18% versus 11%) causing PJI was found in the RA patients compared to OA. There were no bacteria of potential odontogenic origin found in the RA patients, while we found 4% in OA. The bacteria identified in revisions for infection in THRs in patients with RA did not significantly differ from those in OA. Bacteria of oral or dental origin were not found in infected hip joint replacements in RA.

## 1. Introduction

Patients with rheumatoid arthritis (RA) often undergo joint surgery, especially prosthetic joint replacements. In the prebiological agent era (before 2000), 1-2% of patients with RA were estimated to need at least one major joint replacement per year followup [1–4], that is, 25% of the RA patients with 16–20 years of observation [5, 6].

Prosthetic joint infection (PJI) is a serious although infrequent complication to joint replacement surgery. In primary total hip replacements (THR) the risk of deep infection is around 1% [7, 8]. A recent study from the Norwegian Arthroplasty Register (NAR) showed that RA patients had the same overall risk of PJI as patients with osteoarthritis in THR, while the risk of revision for infection more than 6 years postoperatively was higher in RA compared to OA patients [9]. 

In the present paper we seek to evaluate and compare bacterial findings in prosthetic hip joint infections in RA patients versus OA patients, for the following reasons. Firstly, PJIs in patients with RA have been reported to be caused by *Staphylococcus aureus* (*S. aureus*) in as much as 37% [10]. This could be a result of relatively high carriage rates of *S. aureus* in RA patients [11–13]. If this is the case, eradication of nasal *S. aureus* with intranasal mupirocin ointment perioperatively might offer an attractive opportunity for prevention of postoperative prosthetic joint infections caused by *S. aureus* in RA patients undergoing total hip replacement surgery [14–16].

Secondly, RA patients with indwelling hip- or knee-joint prostheses are in some international guidelines considered as exceptional high-risk patients for infections caused by bacteria of potential dental or oral origin. These patients are recommended antibiotic prophylaxis to prevent PJI following bacteremia caused by dental procedures [17–23]. Other guidelines and more recent literature do not mention RA as a high-risk factor and thus do not recommend prophylaxis [24–29] (Table 1). The aims of this study were to evaluate the bacterial findings in PJI among RA patients and compare them to the findings in OA patients with PJI. We particularly focused on the frequency of *S. aureus*. Furthermore, we compared the incidence of PJI caused by microorganisms potentially of oral or dental origin between the groups. This information might contribute in the discussion as to the need for treating the RA patient group different from those with OA, concerning antibiotic prophylaxis.

## 2. Material and Methods

**Figure 1:**
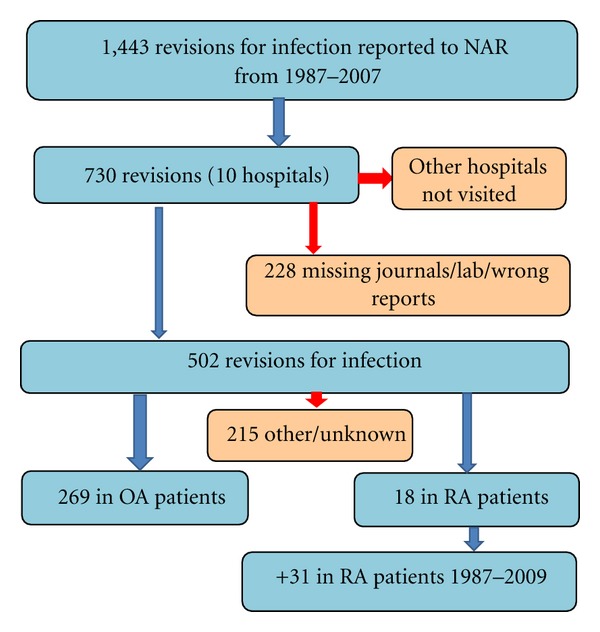
Flow chart showing the inclusion of patients for the study.

The Norwegian Arthroplasty Register includes information on patient identification, the operating hospital, the reason for and the type of primary and revision operations as well as details on the implant type, the fixation method, and the use of antibiotic prophylaxis in each individual case [30, 31]. Primarily included in the present study were all patients having had a PJI leading to a revision, (i.e., surgical exchange or removal of parts of or the whole prosthesis) in the period September 15, 1987 until October 2007. The diagnosis PJI was made by the operating surgeon(s) based on clinical judgement of the pre- and peroperative findings at time of revision surgery, since the registry forms are filled in immediately after surgery, and thus before the analysis of bacterial cultures are finished. During the study period 107,535 primary total hip replacements were registered. One thousand four hundred forty-three revision procedures for infection were reported to the NAR. The ten hospitals performing most revisions for infection, with a total of 730 revisions, were visited (during the year 2009) by one of the authors (H.L). In 228 (mean age at revision: 70 years, 56% females) of the infection episodes the medical records (*n* = 36), bacterial tests (*n* = 96), or the surgical data were incomplete and/or missing (*n* = 96). Thus, bacterial test reports and the surgeons' description of the revision procedure for a total of 502 episodes were systematically reviewed (Figure 1). In 215 infection episodes the diagnosis, that is, indication for primary surgery, was other than OA or RA, and the major diagnostic groups included acute fracture, sequelae after dysplasia, sequelae after fractura colli femoris, M. Pertes/epiphysiolysis, caput necrosis, and unknown diagnosis. 269 infection episodes were seen in 255 patients with OA and in the present study these served as a control group.

In the above mentioned cohort 18 infection episodes (14 patients) were observed in patients with RA as the indication for primary surgery. In the complete NAR database another 23 RA patients that had been revised at 10 hospitals not originally visited, were found and their records were obtained. The hospital with most patients was visited by one of the authors (J. C. Schrama) and the other hospitals were contacted by mail and asked to submit a copy of the medical records. A total of 49 infection episodes in 37 RA patients were thus finally included (Figure 1). Included in our analyses were 292 OA and RA patients (mean age at revision: 72 years, 56% females).

The bacterial findings were obtained from the microbiologic reports in the patient records. Negative cultures (deep and/or biopsy) taken during revision surgery were included and one or more positive cultures were considered as causative for the PJI. We also included bacterial cultures from joint aspiration or blood cultures on the day of revision or 1-2 days before revision surgery. Superficial cultures such as wound swab specimen or swabs from fistulae were excluded. An infection episode (i.e., revision for infection) was considered as a new episode if the patient was assumed clinically free of the former infection and showed unexpected new symptoms of a PJI. Polymicrobial infections, here considered as a separate entity, were defined as infections in which at least two different microorganisms were found. We did not have access to the clinical presentation of the infections, thus no distinction between potentially causative organisms and organisms representing secondary colonisation, could be made. Viridans group streptococci, beta-haemolytic streptococci, *Peptostreptococcus* species and streptococcus-like organisms not further identified, were considered microbes of potential oral or dental origin, as previously described by Berbari et al. [24]. We defined late infections as infections (i.e., revisions for infection) occurring more than 3 months after implantation surgery according to the definition given by Little et al. [32] and Fitzgerald et al. [33]. 

## 3. Statistics

Patient characteristics in the RA and OA group were compared using the chi-square test for categorical variables and the student *t*-test for continuous variables. The proportion of a specific microbe in the RA and the OA group (versus the proportion of all other cases) were compared using the chi-square and the Fisher's exact test. Furthermore, multinomial logistic regression (results not shown in table) was used to investigate the relationship between primary diagnosis and bacterial findings. Results were calculated as odds ratios (OR) with 95% CI comparing the groups CoNS, gram negative bacteria, miscellaneous, mixed flora, and no growth to *S. aureus*. Since a total of 26 patients were registered with more than one revision in the same hip, analyses were also performed based on the first revision only.

Statistical significance was defined as a *P* value less than 0.05. Preceding power analysis showed that, based on Berbari's 10 findings of 37% frequency of *S. aureus* in RA patients our number of observations would achieve 82% power to reveal as statistically significant a 20% difference in group proportions. 

## 4. Results

Seventy per cent of patients with RA were females versus 54% of OA patients (*P* = 0.06, Table 2). At the time of revision RA patients were on average 10 years younger than OA patients (*P* < 0.001, Table 2). The mean time interval from primary surgery until revision for infection was 3.8 years for RA patients and 3.1 years for OA patients (*P* = 0.3, Table 2). *Staphylococcus aureus* was cultured in 9 of the 49 infection episodes (18%) in RA patients and 30 of 269 episodes (11%) in OA patients (*P* = 0.16, Table 3). CoNS tended to be a more frequent finding in patients with OA than in those with RA, although the difference was not statistically significant (18% RA versus 29% OA, *P* = 0.11, Table 3). Using multinomial logistic regression, the odds for culturing CoNS compared to *S. aureus* in RA patients was lower than for OA patients (OR = 0.4, 95% CI: 0.1–1.0, *P* = 0.06) indicating that there were more *S. aureus* compared to CoNS in the RA group. Including only the first revision for infection, the difference was statistically significant (OR = 0.3, 95% CI: 0.1–0.9, *P* = 0.03). Streptococci were cultured in 19 (7%) of the OA patients and in 1 (2%) RA patient (*P* = 0.33). We found no statistically significant difference between the two patient groups with respect to gram negatives (*P* = 0.43), enterococci (*P* = 0.33) and other bacteria (*P* = 1.00) (Table 3). There was however a tendency towards more polymicrobial cultures in the RA group (14 versus 7%, *P* = 0.11). Nor was there any statistically significant difference in the frequency of infections in which no bacteria were detected in the culture (*P* = 0.42). Causative microbes, potentially of oral or dental origin, were found in 12 (4%) of the OA and in none of the RA patients (*P* = 0.13, Table 3).

## 5. Discussion

We found no statistically significant differences in the bacterial findings of infected THRs in RA compared to OA patients. Staphylococci were found in more than half (59.5%) of the positive cultures as reported by others [7, 34].

Mixed or polymicrobial infections had, however, a tendency to be more frequent in the RA group. This finding is in agreement with previous knowledge of wound infections in immune-altered hosts, in whom polymicrobial micro flora is more frequently seen, as for example, in patients with diabetes mellitus [35]. Furthermore, our finding of a high percentage of culture negative infections (31–37%) are caused by prior courses of antimicrobial therapy, inappropriate (handling of the) samples or wrong diagnosis.

Another finding in the present study was a tendency towards a higher frequency of *S. aureus* than, for example, CoNS in RA patients compared to OA patients, although the percentage of infections caused by *S. aureus* was not as high as 37%, reported by Berbari and coworkers, but in their study knee as well as hip replacements were included [10]. In the NAR we have no data on revisions for (early) infections in which no implant parts were exchanged or removed. Thus we may have missed some early infections which are frequently caused by virulent bacteria such as *S. aureus*.

Eight of the nine *S. aureus* infections (analyses not shown) found in the RA patients in the present study were late infections, that is, revised more than 3 months postoperatively. RA patients have previously been shown to be more prone to late infections [9, 36, 37]. These late, potentially blood-borne infections have, according to Maderazo et al. [38], skin and soft tissue as the most common remote sites of infection. *S. aureus* is generally considered unlikely to originate from the mouth and were consequently not included in the group of bacteria of potentially oral or dental origin, in our study. Several authors however advocate the possibility of *S. aureus* originating from the mouth [39, 40]. Particularly acute or chronic dental infections might increase the possibility of culturing Staphylococci species [39].

In the present study we found no significant difference in the occurrence of microbes potentially of oral or dental origin in RA patients compared with OA patients, and the numerical difference between the groups favoured patients with RA among whom no patient had such a microorganism cultured (as opposed to 12 OA patients). Consequently, our findings do not support guidelines that RA patients are high risk patients particularly vulnerable to PJIs caused by microorganisms after transient bacteremia during dental procedures. The findings are in agreement with the existing policy in Norway which has been that RA patients with THR are not given prophylactic antibiotics before dental treatment.

A strength of this study is that it includes data selected from a national registry comprising an entire country (4.8 million inhabitants) over a period of more than 20 years. Data completeness for hip replacements has been shown to be very good, even for revision operations [41]. Although a large RA cohort has been studied previously (200 infection episodes, Berbari et al. [10]), our material is unique in terms of the comparison of microbiology in one of the largest cohorts of RA and OA patients. A drawback is the insufficient statistical power of the study. A *P* value in the nonsignificant range can either reflect an actual lack of difference between the patient groups or that there are too few observations to demonstrate such a difference, if existent. Reported findings should be interpreted with this in mind. Our power calculation was based on the findings of Berbari et al. [10] In their material of 200 infection episodes from the prebiological agents era 37% *S. aureus* was seen in PJIs in patients with RA. We found only 18% *S. aureus* in our material and consequently, there were too few infection episodes in our RA patients to detect a statistically significant difference for *S. aureus* (if present). On the other hand our material is, to our knowledge, one of the largest microbiological materials including and comparing RA and OA patients. Another drawback is the large number of infection episodes with missing or wrong data (228 of 730). Although not analysed, we have no reason to believe that these exclusions represent any kind of selection bias.

Furthermore, patients with PJIs treated solely by conservative means or those treated with limited surgical procedures (not involving removal or exchange of prosthetic parts) were not reported to the registry, and thus were not evaluated in this study.

Finally, we had no information on the patients' medication, which might have had an influence on the microbiology. For instance, immune-modulating antirheumatic medication may increase the risk of infection caused by low-virulent microbes. 

## 6. Conclusion 

We found no differences in the microbiology of infected THRs in RA patients compared to OA patients. There tended to be an increased risk of PJIs caused by *S. aureus* in RA patients, but we did not confirm the high rates of *S. aureus* previously reported in RA. Whether or not there is reason to advise the use of intranasal mupirocine ointment perioperatively as prophylactic strategy against *S. aureus* in PJI's may still be a matter of discussion, but we found no reason to treat the RA group differently in this respect. Furthermore, RA patients seemed less, rather than more, prone to PJIs caused by potentially oral or dental microbes when compared to OA patients. Consequently we cannot, on the basis of our findings, recommend a different policy regarding antibiotic prophylaxis prior to dental treatment in RA patients. RA patients should be individually evaluated with particular emphasis on the patient's comorbidities and medication [25–27]. We advise to continue the common praxis in Norway not giving routinely antibiotic prophylaxis before dental procedures.

## Figures and Tables

**Table 1:** Overview of literature after year 2000 discussing whether rheumatoid arthritis patients are high risk patients for bacteremic prosthetic joint infection after dental treatment and therefore routinely needing antibiotic prophylaxis.

Authors	Year of publication	Country	RA high risk patients, thus antibiotics
ADA and AAOS [17]	2003	USA	Yes
Scott et al. [21]	2005	Australia	Yes
Tong and Theis [22]	2008	New Zealand	Yes
Kotze [18]	2008	South Africa	Yes
Rompen et al. [20]	2008	The Netherlands	Yes
AAOS [23]	2009	USA	Yes
Kuong et al. [19]	2009	Hong Kong	Yes
Seymour et al. [27]	2003	Great Britain	No
Uçkay et al. [29]	2008	Switzerland	No
Blomgren et al. [25, 26]	2009	Sweden	No
Berbari et al. [24]	2010	USA	No

**Table 2:** Patient characteristics.

	RA	OA	*P*
Mean age (years) (SD)*	64 (16)	74 (8)	<0.001
Sex (females%)**	70%	54%	0.06
Mean time to revision (years)*	3.79	3.13	0.3

*In 318 infection episodes, **in the 292 patients.

**Table 3:** Distribution of cultured bacteria at revision surgery in infected THR in RA patients versus OA patients.

	RA (*n* = 49)	OA (*n* = 269)	P*
*Staphylococcus aureus*	9 (18%)	30 (11%)	0.16
Coagulase negative staphylococci	9 (18%)	79 (29%)	0.11
Streptococci	1 (2%)	19 (7%)	0.33
Enterococci	1 (2%)	18 (7%)	0.33
Gram negative bacteria	3 (6%)	10 (3%)	0.43
Others	1 (2%)	10 (3%)	1.00
Polymicrobial flora	7 (14%)	20 (7%)	0.11
No growth	18 (37%)	83 (31%)	0.42
Bacteria potentially of oral or dental origin	0	12 (4%)	0.13

^∗^Chi-square and Fisher's exact test.
